# Prevalence of portal vein thrombosis in non-alcoholic fatty liver disease: a meta-analysis of observational studies

**DOI:** 10.1007/s11239-023-02912-9

**Published:** 2023-12-08

**Authors:** Roberta Stupia, Rosa Lombardi, Filippo Cattazzo, Mirko Zoncapè, Anna Mantovani, Leonardo De Marco, Alessandro Mantovani, Anna Ludovica Fracanzani, David Sacerdoti, Andrea Dalbeni

**Affiliations:** 1 Section of General Medicine C, Azienda Ospedaliera Universitaria Integrata of Verona, University of Verona, P.le Scuro 10, 37134 Verona, Italy; 2 Liver Unit, University and Azienda Ospedaliera Universitaria Integrata of Verona, University of Verona, Verona, Italy; 3 Department of Pathophysiology and Transplantation, Unit of Metabolic and Internal Medicine, University of Milan, Milan, Italy; 4 Section of Endocrinology, Diabetes and Metabolism, University and Azienda Ospedaliera Universitaria Integrata of Verona, University of Verona, Verona, Italy

**Keywords:** Portal vein thrombosis, NAFLD, Liver transplantation cohort, Metabolic diseases, Meta-analysis

## Abstract

Portal vein thrombosis (PVT) is a common complication of cirrhosis as a result of portal hypertension and modification in the hemostatic balance. Accumulating evidence now suggests that patients with non-alcoholic fatty liver disease (NAFLD), especially those with advanced forms, have an increased risk of PVT. Hence, we performed a meta-analysis of observational studies to estimate the overall prevalence of PVT in patients with NAFLD and its advanced forms compared with patients with advanced liver diseases from other etiologies. We systematically searched PubMed, Scopus and Web of Science databases from the inception date to December 30th 2022, using predefined keywords, to identify observational studies. Meta-analysis was performed using random-effects modeling. We included five observational studies for a total of 225,571 patients. Of these, 26,840 (11.9%) patients had NAFLD, whereas the PVT prevalence was 8.5% (n = 2,280). When compared with patients with advanced liver diseases from other etiologies, patients with NAFLD and its advanced forms had a higher risk of prevalent PVT (OR 1.34, 100% CI 1.07–1.67 p < 0,01). The between-study heterogeneity was substantial (I^2^ = 88%). This meta-analysis suggests that compared with patients with advanced liver diseases from other etiologies, patient with NAFLD and its advanced forms had a higher risk of prevalent PVT. Further research is required to understand the complex link between NAFLD/NASH and PVT development.

**Supplementary Information:**

The online version contains supplementary material available at 10.1007/s11239-023-02912-9.

## Highlights

- Meta-analysis of observational studies to estimate the prevalence of PVT in NAFLD patients compared with other etiologies.
- PVT prevalence was 8.5%.
- NAFLD and its advanced forms had a higher risk of prevalent PVT (OR 1.34, 100% CI 1.07–1.67 p < 0,01).
- Further research is required to understand the complex link between NAFLD and PVT.

## Introduction

Portal vein thrombosis (PVT) is a common cirrhosis complication with a prevalence that varies on the basis of diagnosis techniques. For instance, autopsy studies reported a prevalence ranging from 6 to 64% in cirrhotic patients, while studies using ultrasound to diagnose PVT reported a prevalence spanning from 5 to 24% [1].

Different factors, including hypercoagulable state, reduced blood flow in the portal vein, portal hypertension, injury in the vascular endothelium, but also large esophageal varices, previous variceal endoscopic treatment are suggested as potential risk factors for PVT development cirrhotic patients. Moreover, metabolic disorders such as obesity, type 2 diabetes mellitus (T2DM) and hypercholesterolemia lead to a prothrombotic condition [2]. Nonalcoholic fatty liver disease (NAFLD) is the hepatic manifestation of metabolic syndrome and is to date the most common chronic liver disease observed in clinical practice. The NAFLD spectrum includes hepatic steatosis, nonalcoholic steatohepatitis (NASH), NASH-fibrosis, NASH-cirrhosis and hepatocellular carcinoma. NAFLD is now considered as a “systemic disease” which is associated with hepatic and extra-hepatic complications, including fatal and non-fatal cardiovascular events. Interestingly, accumulating evidence suggests that patients with advanced forms of NAFLD have an increased production of pro-thrombotic factors by the liver, like plasminogen activator inhibitor-1 (PAI-1) or factor VIII and reduced levels of anticoagulant factors [3], consequent to the chronic inflammation with oxidative injury, necrosis, and apoptosis [4–6]. Moreover, alterations in platelets number and activity are usually found in NAFLD patients, with high levels of pro-fibrotic and pro-inflammatory activities, fostering atherothrombosis and possibly fibrogenesis [7, 8]. In addition, platelets in NAFLD seems to express the leptin receptor, a hormone which increased levels have been associated with a hypercoagulability state, arterial thrombosis and PVT development in non-cirrhotic NAFLD patients. [9]

At present, evidence about the association of NAFLD and its advanced forms with the risk of PVT are still inconclusive. In addition, available studies mainly focus on the onset was pre-transplant PVT in liver transplant (LT) recipients, showing a negative impact of NAFLD etiology sustaining cirrhosis requiring LT [10]. Herein, we have conducted a meta-analysis of observational studies to evaluate the association between NAFLD and its advanced forms and risk of PVT when compared with patients with advanced liver diseases from other etiologies.

## Materials and methods

Registration of protocol the protocol of this systematic review and meta-analysis was registered in advance in Open Science Framework database (10.17605/OSF.IO/F2CR9).

### Data sources and searches

We performed a systematic review and meta-analysis in accordance with the Preferred Reporting Items for Systematic Reviews and Meta-Analyses (PRISMA) guidelines (http://www. prisma- statement.org.) (see PRISMA 2020 flow diagram). Given that the included studies were observational in design, we also followed the reporting items proposed by Meta-analysis of Observational Studies in Epidemiology guidelines for the meta-analysis of these studies [11].

We systematically searched PubMed, Scopus and Web of Science databases from the beginning date to 30 December 2022 using the following keywords: ‘nonalcoholic fatty liver disease’ OR ‘NAFLD’ OR ‘NASH’ OR ‘MAFLD’ OR ‘metabolic syndrome’ AND ‘portal vein thrombosis’ in order to identify observational studies. Additionally, we reviewed all references from relevant original papers and review articles in order to identify further eligible studies that are not covered by the original database searches.

For all retrieved studies, we extracted the following information: authors, publication year, country, study design, sample size, number of NAFLD/NASH patients, number of patients with PVT in patients with and without NAFLD, outcomes of interests (adjusted odds ratios (ORs), 95% confidence intervals (CIs) for the association between NAFLD/NASH and risk of PVT). Additional information, such as age, sex, body mass index, and prevalence of diabetes mellitus in the patient population included in our meta-analysis, was also extracted.

### Study selection

Eligible observational studies were included if they met the following criteria:

1. Observational studies examining the association between NAFLD/NASH and risk of PVT;
2. Studies reporting odd ratios and 95% CI values for the outcome measure of interest;
3. The diagnosis of NAFLD/NASH was based on liver biopsy, imaging techniques or International Classification of Diseases, 9th Revision (ICD-9) or ICD-10 codes, in the absence of significant alcohol consumption and chronic viral hepatitis;
4. The diagnosis of the outcomes of interest was based on imaging techniques, ICD-9/ICD-10 codes.

Study participants included in the meta-analysis were of either sex without any restriction in terms of race, ethnicity or comorbidities.

Criteria for exclusion of the selected observational studies from this meta-analysis were as follows:

1. Animal trials, case reports, letters to the editor, guidelines, reviews or meta-analyses, studies with inadequate data on the outcomes of interest;
2. Studies where NAFLD/NASH diagnosis was based exclusively on serum liver enzyme levels or other surrogate markers of NAFLD (e.g., fatty liver index);
3. Studies which did not specifically report any OR and 95% CI for the outcome measure of interest;
4. Studies without an appropriate control group;
5. Studies conducted in pediatric population (< 18 years old).
6. Malignancy except for hepatocellular carcinoma (as reported in each studies).

### Data extraction and quality assessment

Two investigators (RS and AD) independently examined all titles and abstracts as well as acquired full texts of potentially relevant papers. Working independently and in duplicate, they read the papers and determined whether they met inclusion criteria. Discrepancies were resolved by consensus, in consultation with a third author (RL). For all studies, we extracted information on study design, sample size, study country, population characteristics, modality of NAFLD diagnosis, modality of PVT diagnosis, and confounding factors included in multivariable regression analyses. In the case of multiple publications, we included the most up-to-date or comprehensive information. We did not contact any corresponding author of the eligible studies in order to obtain additional information for the meta-analysis.

Two authors (RS and AD) independently assessed the risk of bias. Any discrepancies were addressed by a re-evaluation of the original article by a third author. Since all the included studies were non-randomized and had a cross-sectional design, the Newcastle-Ottawa Scale (NOS) adapted for cross-sectional studies was used to judge study quality. Specifically, the NOS uses a star system (with a maximum of nine stars) in order to evaluate a study in three specific domains: selection of participants, comparability of study groups and the ascertainment of outcomes of interest.

We considered studies that received a score of eight to ten stars to be at low risk of bias, studies that scored six or seven stars to be at medium risk and those that scored five or less to be at high risk of bias (supplementary Table S1).

### Data synthesis and analysis

The primary outcome measure of this systematic review and meta-analysis was the prevalence of PVT in patients with NAFLD and its advanced forms when compared with patients with advanced liver diseases from other etiologies. The ORs with their 95% CIs were considered as the effect size for all eligible studies. When studies had several adjustment models, we extracted those that reflected the maximum extent of adjustment for potentially confounding risk factors. The adjusted ORs of all eligible observational studies were then pooled, and an overall estimate of effect size was calculated using the REML (Restricted Maximum-Likelihood) method. We used this method because it produces an unbiased, nonnegative estimate of the between-study variance. Visual inspection of the forest plots was used to investigate the possibility of statistical heterogeneity. Statistical heterogeneity was also assessed by the I^2^ statistics. Give that the eligible studies were less than 10, we did not perform funnel plot and the Egger’s regression test to assess the potential publication bias. We assessed for possibly excessive influence of individual studies using a meta-analysis influence test that eliminated each of the included studies at a time.

All statistical tests were two sided and used a significance level of p < 0.05. We used R software R version 4.1.0 (2021-05-18) for all statistical analyses. Specifically, meta (version 6.0–0) and metafor (version 3.8-1) packages.

## Results

We initially identified 13 potentially relevant observational studies from the three large electronic databases, from prior to 30 December 2022 (date of the last research). Of these, 8 studies were then excluded for inadequate data regarding the outcomes and/or for unsatisfactory inclusion criteria. The observational studies excluded at the eligibility step of PRISMA diagram are reported in the Supplementary Table S2.

Based on this selection, 5 unique, observational studies, for a total of 225,571 adult individuals (mean age 53.2 ± 5.6 years, BMI 28.4 ± 1.2 Kg/m^2^, percentage of men 65%, percentage of patients with NAFLD/NASH 11.9%, percentage of patients with diabetes mellitus 16%) were included in the final analysis.

The principal characteristics of the selected studies are reported in Table 1. In particular, 4 studies were performed in USA and a study was carried out in Iran. All eligible studies used ultrasonography to detect NAFLD and PVT. For all studies, control group was characterized by individuals with chronic liver disease due to alcohol, virus or autoimmunity.

**Table 1:** Eligible observational studies [15–19] assessing the association between NAFLD and the risk of prevalent portal vein thrombosis (PVT) (ordered by publication year)

Author	Year	Country	Sample size	Number of men	Mean age (years)	Mean body mass index (Kg/m^2^)	Number of patients with diabetes mellitus	NAFLD diagnosis	Control group etiology	Number of patients with PVT	Number of patients with NAFLD	Number of patients with PVT in NAFLD group	Adjusted odds ratio	Lower CI	Upper CI
Ghabril	2016	USA	48,570	32,992	56	28.7	10,766	US	Alcohol / HCV	3321	2932	332	1.07	1.54	1.95
Stine	2017	USA	35,072	23,846	60	29.5	7909	US	Alcohol / autoimmune, / cholestatic / HBV / HCV	2624	3251	394	2.11	1.6	2.76
Montenovo	2018	USA	128,160	82,653	54.6	29.2	15,530	US	Acohol	5009	17,008	995	1.1	1.01	1.15
Eshraghian	2018	Iran	1007	644	42	25.5	252	US	Autoimmune / HBV / HCV	174	373	74	1.36	1.08	1.72
Molinari	2021	USA	12,762	6199	55	29.8	2565	US	Cholestatic / alcohol / HBV / HCV / HCC	1011	3276	485	1.41	1.22	1.62

**Fig. 1:**
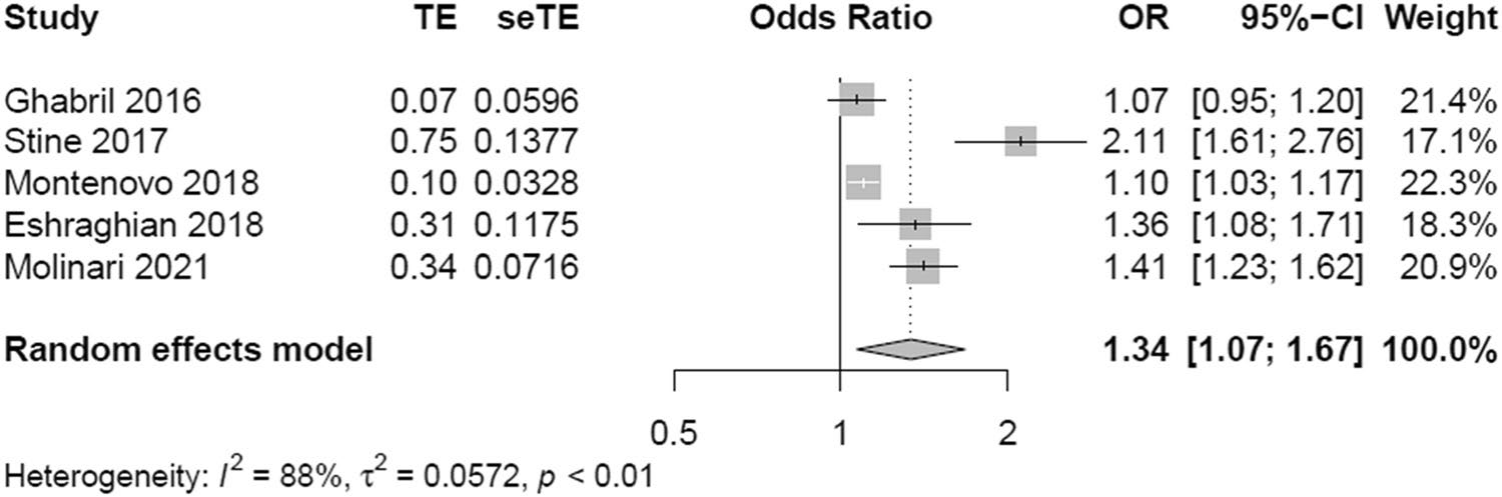
Forest plot reporting the association between NAFLD and the risk of prevalent portal vein thrombosis (PVT)

**Fig. 2:**
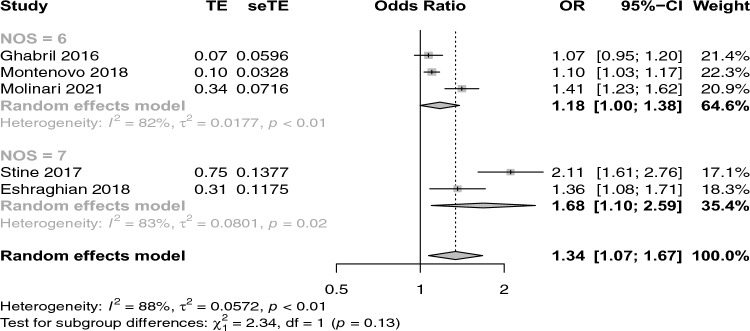
Forest plot reporting the association between NAFLD and the risk of prevalent portal vein thrombosis (PVT), stratifying the studues by NOS scale

**Fig. 3:**
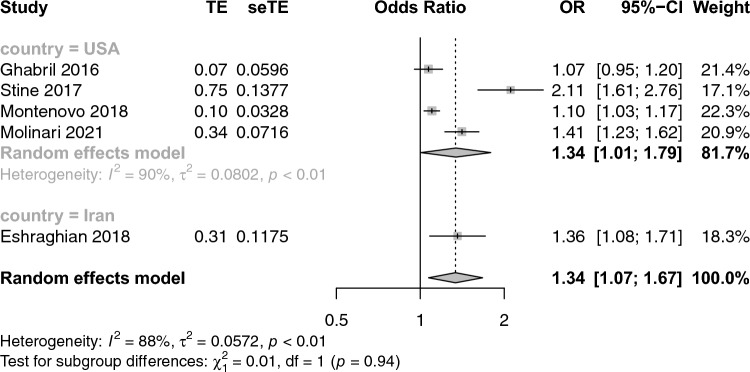
Forest plot reporting the association between NAFLD and the risk of prevalent portal vein thrombosis (PVT), stratifying the studies by country

Figure 1 shows the forest plot reporting the association between NAFLD and its advanced forms and the risk of prevalent PVT. Interestingly, patients with NAFLD and its advanced forms had a higher risk of prevalent PVT compared with patients with advanced liver diseases from other etiologies. (n = 5; random effects odd ratio 1.34, 95% CI 1.07–1.67; I^2^ = 88%). Stratifying the eligible studies by study country, the strength of the association between NAFLD and its advanced forms and risk of PVT was substantially similar among studies performed in USA (n = 4; random effects odd ratio 1.34, 95% CI 1.01–1.79; I^2^ = 90.2%) and in Iran (random effects odd ratio 1.36, 95% CI 1.08–1.71; I^2^ = not determinated) (Fig. 2). Stratifying the eligible studies by NOS scale, the strength of the association between NAFLD/NASH and risk of PVT was higher in the studies receiving 7 stars (Fig. 3).

As summarized in Supplementary Table S1, all the studies received six or seven stars indicating an overall medium risk of bias. Syntax used through database searching on PubMed, Scopus or Web of Science is reported in Supplementary Table S3.

## Discussion

Our meta-analysis including five eligible studies demonstrated a prevalence of PVT in the NAFLD population of about 8% and, most importantly, that patients with NAFLD and its advanced forms have a pooled 1.34-fold increased risk of prevalent PVT, when compared to advanced liver diseases from other etiologies. At present, available data are not still conclusive on this topic and, first of all, focus on the onset of pre-transplant PVT in LT recipients, thus showing a negative impact of NAFLD etiology sustaining cirrhosis requiring LT on the thrombosis complication. This evidence provids additional support for the assertion that NAFLD and its advanced forms predispone to a prothrombotic state [10–12]. Along with cirrhotic complications, another small study including prospectively 94 NAFLD non-cirrhotic patients, showed the occurrence of PVT in 8% of the cohort even without advance liver disease, especially in obese subjects and those with increased leptin levels^9^. Nevertheless, this study does not evaluate any association with histological features of severity of NAFLD, and no other study includes patients with NAFLD without cirrhosis, so that the question of whether the increased risk for PVT is restricted to patients with NASH or applies to all patients with NAFLD remains largely unsolved so far. If on the one hand, studies on this issue are scarce, on the other hand meta-analysis evidence is even less pronounced. In fact, only one meta-analysis by Li et al. including 22 studies aiming at defining the association of PVT with cirrhosis of different etiologies, quantified the magnitude of the association between metabolic alterations and PVT and highlighted a significant association with T2DM and dyslipidemia. When focusing only on 4 studies (n = 3,385,821 patients) reporting a NAFLD etiology for cirrhosis, the authors showed a significant association between NAFLD and PVT, with a pooled random effects OR 1.61 (95% CI 1.34–1.95) [13]. This risk was slightly higher compared to those found in our analysis. However, this study did not specifically focus on NAFLD, but on metabolic abnormalities and reported a transversal association between PVT and liver disease without prompting the drive of a final causative relationship. In addition, in this analysis, a statistically significant between-study heterogeneity was also observed (I^2^ = 75%) [14], similar to those found in our study. Another small meta-analysis including 3 retrospective studies with cirrhotic patients from different etiologies, demonstrated an independent association between NAFLD and PVT (random effects OR 2.12, 95% CI 1.45–3.09). However, the solidity of reports was weak as two articles were conference abstract and only one article was an original one [13].

Our meta-analysis has some important limitations that are strictly inherent to the design of the included studies. First, the cross-sectional design of the elibible studies does not allow establishing any temporal and causal association between NAFLD and PVT. Second, although the eligible studies adjusted their results at least for age, sex, smoking, obesity and T2DM, the possibility of residual confounding by some unmeasured (or unknown) factors cannot be ruled out. For example, the majority of the eligible studies reported incomplete adjustments for some important risk factors, such as waist circumference, drug use, procoagulant state. Moreover, in most of the studies, associated oncological diseases were excluded (except for HCC) and probably also prothrombotic states considering that the cohort analysed came from transplant cohorts. However, it is not possible to exclude with certainty any conditions conducive to thrombotic states. However, the NOS quality scale of the eligible studies suggested an overall medium risk of bias. Third, another limitation of the meta-analysis is that the eligible studies used imaging techniques, but none of them used liver biopsy, which is the reference standard for diagnosing and staging liver disease. In addition, almost all studies included Caucasian populations, thus limiting the generalization of results to other ethnicities and pre-transplant population, so selecting a category of patients with advanced liver disease. Fourth, our results should be interpreted with caution, because of the high heterogeneity across the eligible studies. Notably, specific subanalysis by study country and NOS did not substantially reduce the high heterogeneity we observed. In addition, we were unable to perform additional sub-analyses or even meta-regressions (given the relatively small number of the studies included) to further assess the causes of heterogeneity. Therefore, speculatively, we believe that heterogeneity in our meta-analysis may be mainly due to different inclusion criteria of patients, limitation of ultrasound in diagnosing NAFLD and PVT, as well as to different covariates used in the eligible studies.Not with standing these limitations, this is the first uptodate meta-analysis of observational studies available in literature, which specifically focus its attention on the association between NAFLD and PVT. It is beyond the scope of this meta-analysis to discuss in depth the putative underlying mechanisms by which NAFLD and its advanced forms may contribute to the development of PVT. We confirm this association possibly sustaining the higher hypercoagulability state of NAFLD compared to other etiologies. Whether NAFLD is associated with an increased risk of PVT simply as a consequence of metabolic comorbidities which characterize the liver disease, or if NAFLD, especially in its advanced forms, may contribute to its development remains an open question.

In conclusion, our meta-analysis of observational studies reported that patients with NAFLD and its advanced forms have an increased risk of prevalent PVT, when compared to advanced liver diseases from other etiologies. Our results may suggest that clinicians should have particularly attention on the procoagulant state in patients with NAFLD, especially those with advanced forms.

However, further larger studies, possibly prospective, are warranted to confirm this evidence.

### Supplementary Information

Below is the link to the electronic supplementary material.

Supplementary material 1 (DOCX 21.0 kb)

Supplementary material 2 (DOCX 31.5 kb)

Supplementary material 3 (DOCX 43.0 kb)

## Data Availability

All data are reported in the draft and supplementary material.
