# Supplementary material for: Prevalence of portal vein thrombosis in non-alcoholic fatty liver disease: a meta-analysis of observational studies
Source: J Thromb Thrombolysis. 2023 Dec 8;57(2):330–6. doi: 10.1007/s11239-023-02912-9 (PMC10869434; doi:10.1007/s11239-023-02912-9)
Supplement: Supplementary file 1 — Supplementary material 1 (DOCX 21.0 kb) [file 11239_2023_2912_MOESM1_ESM.docx]

**Supplementary Table S1. The Newcastle-Ottawa Scale (NOS) for assessing the quality of studies in meta-analyses**

| **Author, year** | **Selection** | | | | **Comparability** | **Outcome** | | **Total Quality Score** |
| --- | --- | --- | --- | --- | --- | --- | --- | --- |
| Montenovo/2018 | * | * | * |  | * | * | * | 6 |
| Eshraghian/2018 | * | * | * | * | * | * | * | 7 |
| Stine/2017 | * | * | * | * | * | * | * | 7 |
| Molinari/2021 | * | * | * |  | * | * | * | 6 |
| Ghabril/2016 | * | * | * |  | * | * | * | 6 |

**Supplementary Table S2**. Studies excluded at the eligibility step of PRISMA diagram

| **Author, year** | **PMID** | **Reason(s) for exclusion** |
| --- | --- | --- |
| Abdel-Raziz, 2021 | 33996858 | Unsatisfactory study design |
| Agbim U, 2019 | 30091296 | Unsatisfactory study design |
| Karvellas CJ, 2017 | 28233745 | Unsatisfactory inclusion criteria |
| Villa, 2012 | 22819864 | Unsatisfactory outcome |
| Wan, 2017 | 29137043 | Unsatisfactory outcome |
| Bureau, 2016 | 26334577 | Unsatisfactory inclusion criteria |
| Hernandez-Conde, 2019 | 31393183 | Unsatisfactory study designe |

**Supplementary Table S3.** Syntax used through database searching on PubMed, Scopus or Web of Science.

**PubMed <up to December 30, 2022>**

| #1 | Search “nonalcoholic fatty liver disease” AND “portal vein thrombosis” | 53 |
| --- | --- | --- |
| #2 | Search “NAFLD” AND “portal vein thrombosis” | 47 |
| #3 | Search “NASH” AND “portal vein thrombosis” | 56 |
| #4 | Search “MAFLD” AND “portal vein thrombosis” | 47 |
| #5 | Search “metabolic syndrome” AND “portal vein thrombosis” | 25 |

**Web of Science <up to December 30,2022>**

| #1 | Search “nonalcoholic fatty liver disease” AND “portal vein thrombosis” | 68 |
| --- | --- | --- |
| #2 | Search “NAFLD” AND “portal vein thrombosis” | 68 |
| #3 | Search “NASH” AND “portal vein thrombosis” | 82 |
| #4 | Search “MAFLD” AND “portal vein thrombosis” | 1 |
| #5 | Search “metabolic syndrome” AND “portal vein thrombosis” | 181 |

**Scopus <up to December 30, 2022>**

| #1 | Search “nonalcoholic fatty liver disease” AND “portal vein thrombosis” | 217 |
| --- | --- | --- |
| #2 | Search “NAFLD” AND “portal vein thrombosis” | 96 |
| #3 | Search “NASH” AND “portal vein thrombosis” | 206 |
| #4 | Search “MAFLD” AND “portal vein thrombosis” | 4 |
| #5 | Search “metabolic syndrome” AND “portal vein thrombosis” | 165 |
