# Supplementary material for: Prevalence of portal vein thrombosis in non-alcoholic fatty liver disease: a meta-analysis of observational studies
Source: J Thromb Thrombolysis. 2023 Dec 8;57(2):330–6. doi: 10.1007/s11239-023-02912-9 (PMC10869434; doi:10.1007/s11239-023-02912-9)
Supplement: Supplementary file 3 — Supplementary material 3 (DOCX 43.0 kb) [file 11239_2023_2912_MOESM3_ESM.docx]

**Identification of studies via databases and registers**

Records removed *before screening*:

Duplicate records removed (n = 78 )

Records marked as ineligible by automation tools (n = 36 )

Records removed for other reasons (n = 1 )

Records identified from*:141

Databases (n = 141 )

**Identification**

Records excluded**

(n = 13 )

Records screened

(n =26 )

**Screening**

Reports excluded:

Irrelevant outcome (n = 6)

Not NAFLD patients (n = 2)

Reports assessed for eligibility

(n =13 )

Studies included in review

(n =5)

**Included**

*Consider, if feasible to do so, reporting the number of records identified from each database or register searched (rather than the total number across all databases/registers).

**If automation tools were used, indicate how many records were excluded by a human and how many were excluded by automation tools.

*From:*  Page MJ, McKenzie JE, Bossuyt PM, Boutron I, Hoffmann TC, Mulrow CD, et al. The PRISMA 2020 statement: an updated guideline for reporting systematic reviews. BMJ 2021;372:n71. doi: 10.1136/bmj.n71

For more information, visit: <http://www.prisma-statement.org/>
